# Correlation between sleep and multimorbidity in community-dwelling older adults in Hunan Province: a cross-sectional study

**DOI:** 10.3389/fpubh.2025.1514524

**Published:** 2025-04-25

**Authors:** Manman Su, Yang Zhou, Wenhui Chen, Yanping Liu

**Affiliations:** ^1^Teaching and Research Section of Clinical Nursing, Xiangya Hospital, Central South University, Changsha, China; ^2^Operating Room, Xiangya Hospital, Central South University, Changsha, China; ^3^Department of Nursing, Xiangya Hospital, Central South University, Changsha, China; ^4^Department of Child Rehabilitation, The Maternal and Child Health Hospital of Hunan Province, Changsha, China

**Keywords:** sleep, multimorbidity, chronic disease, older adults, China

## Abstract

**Background:**

The aim of this study was to provide global estimates of the prevalence of sleep quality and chronic diseases and to analyze the correlation between sleep and multimorbidity in community-dwelling older adults (≥65 years) in China.

**Methods:**

This is a cross-sectional study and a stratified multistage random sampling method was used to collect data on demographic characteristics, the Pittsburgh Sleep Quality Index (PSQI), and assessment of chronic conditions. Chi-squared tests, *t*-tests, Wilcoxon rank sum test and analysis of variance were used to test the correlation between sleep and multimorbidity.

**Results:**

Of the 1,173 community-dwelling older adults enrolled, the mean total PSQI score was 7.25 ± 4.23. Of these, 588 (50.1%) had a total PSQI score >7 (with poor sleep quality) and 920 (78.4%) had chronic diseases. In addition, 43.8% (403/920) had a single chronic disease and 56.2% (517/920) had multimorbidity. A combination of two and three chronic conditions dominated the pattern of multimorbidity among community-dwelling older adults. Overall, the prevalence of poor sleep quality with multimorbidity among community-dwelling older adults in the community was 57.6% (298/517). The prevalence of poor sleep quality in older adults with multimorbidity was 1.30 times higher than in those without multimorbidity (RR = 57.6%/44.2% = 1.30). The prevalence of poor sleep quality increased with the number of chronic conditions, and women had a higher prevalence of poor sleep quality than men. *T*-test and analysis of variance showed statistically significant differences in all seven components of the PSQI between those with and without multimorbidity and different numbers of chronic conditions (*p* < 0.05).

**Conclusion:**

Community-dwelling older adults with multimorbidity were more likely to have sleep problems. The number of chronic conditions also had an additive effect on sleep problems, and women reported poorer sleep quality than men. One of the most challenging aspects of falling asleep was for participants with multimorbidity.

## 1 Introduction

Globally, every country is experiencing growth in both the size and proportion of older adults in the population. Between 2015 and 2050, the proportion of the world’s population over 60 years will almost double from 12 to 22% (1). Since entering the aging society in 2000, the aging of China’s population has continued to deepen (2). Data from the Seventh Population Census show that the degree of aging is on the rise. By the end of 2020, China’s senior citizens over 60 years has reached 264 million, accounting for 18.7% of the total population (3). Increasing health problems associated with aging will seriously affect the healthy life expectancy and quality of life of seniors, and will also place a heavy economic burden on families and society (4).

Sleep is an active periodic biological phenomenon that is necessary for human survival and is one of the most important human behaviors, accounting for approximately one third of human life (5). Sleep quality is a measure of how a person feels about being energetic, active, and ready for a new day (6). Sleep quality is closely related to the level of health, well-being and quality of life of human psychological and physiological functions (7). Good sleep quality protects the psychological and somatic functional status of older adults (8). Sleep quality is essential for health, but declines with age (9). The World Health Organization has identified poor sleep quality as a public health problem that increases the risk of death (10).

Multimorbidity is an age-related clinical chronic disease syndrome, defined as the presence of two or more chronic diseases at the same time (11). The prevalence rate of chronic multimorbidity among people aged 40 to 60 years in Singapore was 41.7% (12). The detection rate of chronic multimorbidity in middle-aged and older adults in China was 55.7% (13). In seniors, the phenomenon of “one body and multiple diseases” is more common, with the rate of coexistence of multiple diseases as high as 55 to 98% (14). In South America, the overall prevalence of multimorbidity was 33.1%, with 2, 3, 4, 5 and 6 chronic conditions found in 19.9, 9.1, 2.6, 1.1 and 0.4% of the population, respectively (15). Compared with those with one or no chronic condition, people with multimorbidity have a higher risk of death, poorer quality of life, and greater financial burden (5). Multimorbidity is an increasing public health concern as the world’s population continues to grow at an accelerated rate.

Current research shows that abnormal sleep duration and poor sleep behavior (including sleep duration and sleep quality) are associated with several chronic conditions, such as hypertension (16), diabetes (17), hyperlipidemia (18), osteoarthritis (19), obesity and affective symptoms (e.g., anxiety, depression). Although the correlation between sleep behavior and single chronic diseases is well established, researchers have paid more attention to the correlation with multimorbidity. Recent evidence from 46 low-and middle-income countries, excluding China, Comoros, Republic of Congo, Ivory Coast, India, and Russia, confirmed that physical multimorbidity was positively associated with sleep problems in adults (20). Results from the UK found that short sleep duration at ages 50, 60, and 70 was associated with risk of chronic disease and subsequent multimorbidity but not with progression to death (21). A study of Brazilians aged ≥ 18 years showed a higher prevalence of multimorbidity in self-reported sleep problems (22). Data from the Chinese Longitudinal Healthy Longevity Survey (2014 wave) (23) highlighted the importance of normal nightly sleep duration and good sleep quality in preventing multimorbidity. A survey in Shanxi Province, China, found that older adults with low socioeconomic status (SES) and poor sleep quality had the highest risk of the prevalence of multimorbidity (24). However, the prevalence, pattern and demographic factors of multimorbidity and its correlation with sleep in older adults remain unclear.

Given the importance of sleep as a major public health issue in a given population and the clinical significance of multimorbidity, the aim of the present study was to provide global estimates of the prevalence of sleep quality and chronic disease conditions among community-dwelling older adults in China. In addition, the correlation between sleep and multimorbidity was analyzed to provide a reference for the prevention and control of chronic disease comorbidity.

## 2 Materials and methods

### 2.1 Study design

A cross-sectional community-based study design was adopted from March 1, 2022 to May 30, 2022. The study was conducted in two cities of Changsha and Xiangtan in the Hunan Province, China.

### 2.2 Setting

Two cities (states) were randomly selected from 14 cities and states in Hunan Province, Changsha City and Xiangtan City. Participants were all regular residents of both cities.

### 2.3 Participants

#### 2.3.1 Inclusion and exclusion criteria

The inclusion criteria were as follows: (1) ≥ 65 years of age; (2) All were permanent residents of Hunan Province (residence time > 6 months); (3) Normal communication ability; (4) Voluntary participation and signed informed consent.

The exclusion criteria were as follows: (1) With severe organic mental disorders or undergoing psychiatric treatment; (2) With cognitive impairment.

#### 2.3.2 Sample size calculation

The minimum sample size was calculated by the equation $n={\left(\frac{u_{а/2}\sigma }{\delta }\right)}^{2}$ (25), when *α* = 0.05,$u_{0.05/2}$=1.96, $u_{0.05/2}\sigma$=1.37, δ=0.1, which was equal to 721.02 ≈ 722. If *n* is the required sample size and *d* is the dropout rate as per formula, then adjusted sample size *N_1_* is obtained as *N_1_* = *n*/(1-*d*). Considering a 20% non-responders rate, the target sample size was *N_1_* = 721.02 / (1–0.20) = 901.275 ≈ 902. Finally, 1,173 valid questionnaires were collected in this study.

### 2.4 Sampling procedure

**Figure 1:**
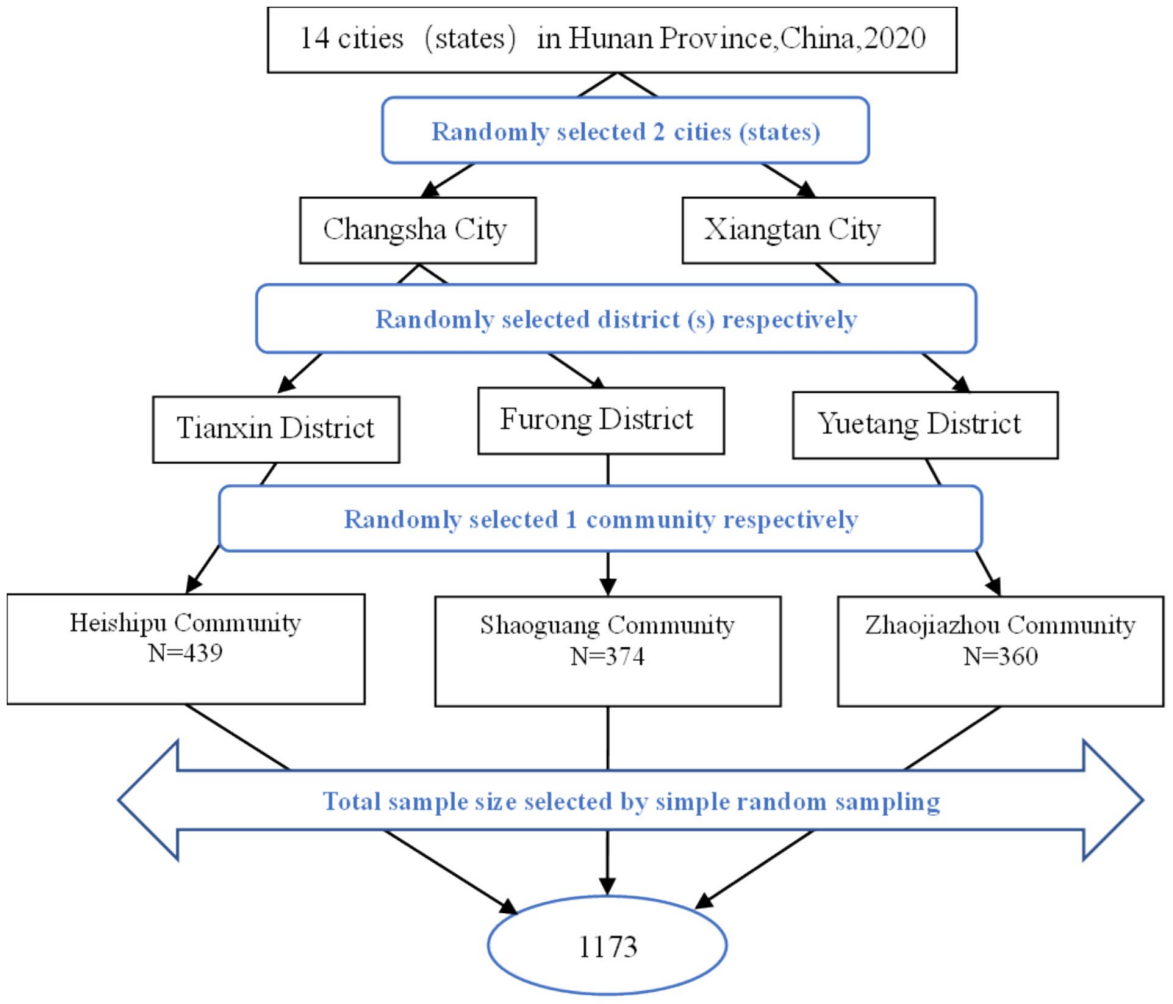
Flowchart for screening the subjects (*N* = 1,173).

The stratified multi-stage random sampling method was used to collect the samples. First, the 14 cities (states) were numbered from 1 to 14, and two cities (states), Changsha City and Xiangtan City, were randomly selected from Hunan Province. Then, the districts administered by the two cities are numbered starting from 1 to 50, two districts, Tianxin District and Furong District, were randomly selected from Changsha City, and Yuetang District was randomly selected from Xiangtan City by further stratification. Finally, the communities in the selected districts are numbered from 1 to 200, three communities (not retirement homes), Heishipu Community (*N* = 439), Shaoguang Community (*N* = 374), and Zhaojiazhou Community (*N* = 360) were randomly selected from the selected districts. Figure 1 shows the flowchart for screening the subjects in this study.

### 2.5 Demographic characteristics

Data included sociodemographic characteristics (e.g., gender, age in years, marital status, educational level, number of children, pre-retirement occupation, monthly personal income, and religion), body mass index (BMI), and lifestyle habits (e.g., smoking and physical activity).

### 2.6 Pittsburgh sleep quality index (PSQI)

Sleep quality among older adults was assessed using the Chinese version of the Pittsburgh Sleep Quality Index (PSQI), developed by Buysse et al. (26) in 1989 and translated by Liu and Tang (27) in 1996. PSQI is a self-rated questionnaire that assesses sleep quality over the past month. It consists of 19 self-rated items and 5 other-rated items, of which the 19th self-assessment question and 5 other-rated questions are not part of the calculation of the PSQI score. It contains 18 items grouped into seven components: subjective sleep quality, time to fall asleep, sleep duration, sleep efficiency, sleep disturbance, hypnotic medication, and daytime dysfunction. Each component score ranges from 0 to 3, and the total score ranges from 0 to 21. And the higher the score, the worse the quality of sleep. The Cronbach alpha of the scale was 0.842 and the split-half reliability was 0.866 (27). Participants with a global PSQI score greater than 7 were defined as having poor sleep quality in this study.

### 2.7 Chronic condition assessment

Self-reported chronic diseases were measured by a multiple choice question, which referred to chronic non-communicable diseases. A total of 9 chronic conditions of all human systems (e.g., hypertension, hyperlipidemia, diabetes, stroke or coronary heart disease, respiratory diseases, Alzheimer’s disease, urinary diseases, and arthritis) were coded according to the International Classification of Diseases (ICD-10). Multimorbidity was defined as having at least two chronic conditions, in line with previous definitions. The number of chronic conditions was also classified as 0, 1, 2, 3, 4 and ≥5 conditions.

### 2.8 Data collection procedure

The study protocol was approved by the Medical Ethics Committee of Xiangya Hospital of Central South University (reference number: 201909818). Prior to the investigation, investigators received uniform training on objectives, methodology, tools, ethical issues, data collection, and some considerations. Subsequently, investigators who received unified training visited different communities. The purpose, benefits, and risks of participating in the research were explained to potential participants. All participants provided written informed consent prior to participating in the survey. Participants were also informed that their participation was voluntary and that they had the right to withdraw at any time. Information was obtained through face-to-face interviews conducted by trained interviewers. The questionnaire was subjected to standard translation procedures to ensure comparability. Each questionnaire took about 15–20 min to complete. After completing each questionnaire, the investigator will check the questionnaire on the spot to ensure the integrity and accuracy of the data. Initially, 1,200 older adults consented to participate in the study and received the questionnaires. Of these, 27 were ultimately excluded from the analysis due to incomplete or missing data. In total, 1,173 questionnaires were eligible for data analysis in the study, resulting in a response rate of 97.8%.

### 2.9 Statistical analysis

The statistical analysis was performed with IBM SPSS Statistics 23.0. Continuous variables (such as age, BMI, and PSQI scores) are presented as mean ± standard deviation (SD) and categorical variables (such as gender, age group, BMI group, marital status, educational level, number of children, pre-retirement occupation, monthly personal income, religion, smoking, physical activity, and number of chronic conditions) as absolute frequencies (percentages). The prevalence of poor sleep quality in the number of chronic conditions was tested by Chi-squared tests. The Wilcoxon rank sum test was used to compare the number of chronic conditions in older adults with and without poor sleep quality. The PSQI total score and dimension scores between multimorbidity and non- multimorbidity were analyzed by two sample *t*-tests. The difference in total PSQI scores and dimension scores among chronic conditions was compared by variance analysis. All *p*-values < 0.05 were considered statistically significant (two-tailed).

## 3 Results

### 3.1 Overall status of sleep and multimorbidity in community-dwelling older adults

#### 3.1.1 Status of PSQI in community-dwelling older adults

Of the 1,173 community elders enrolled, the mean total PSQI score was 7.25 ± 4.23. Among the study subjects, 588 participants had a total PSQI score >7 (with poor sleep quality), accounting for 50.1% of the total.

#### 3.1.2 Prevalence and pattern of multimorbidity in community-dwelling older adults

**Figure 2:**
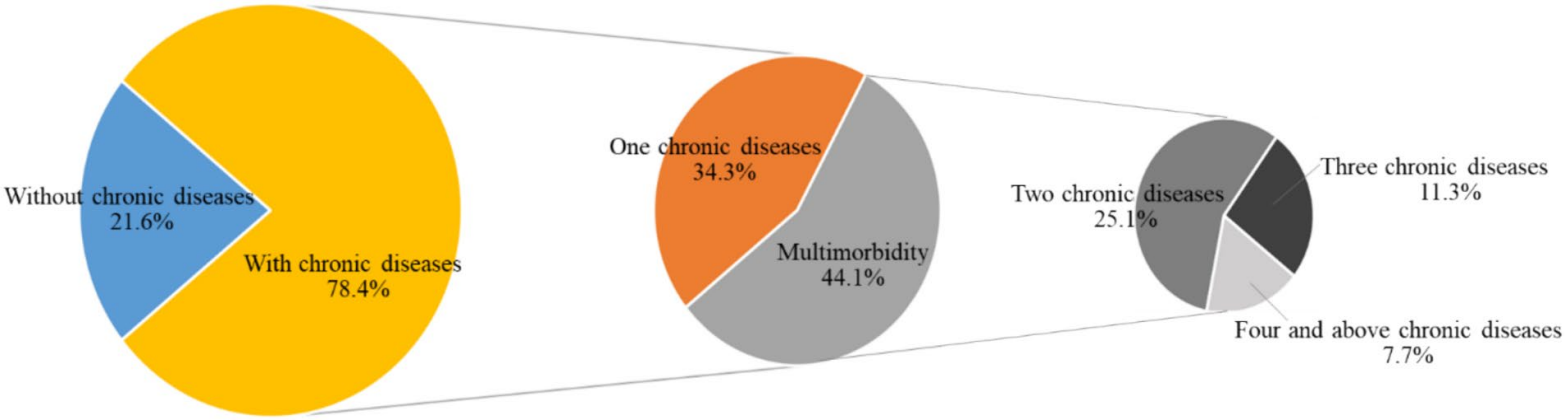
The prevalence of multimorbidity in community-dwelling older adults (*n* = 1,173).

Of the 1,173 people, 253 (21.6%) had no chronic diseases and 920 (78.4%) had chronic diseases. Of 920 people with chronic diseases, 43.8% (403/920) had one chronic disease and 56.2% (517/920) had multimorbidity. Of the 517 community-dwelling older adults with multimorbidity, 56.9% (294/517) had two chronic diseases, 25.7% (133/517) had three chronic diseases, and 17.4% (90/517) had four and more chronic diseases. The prevalence of multimorbidity is shown in Table 1 and Figure 2. And the pattern of multimorbidity among community-dwelling older adults was dominated by a combination of binary and ternary chronic diseases.

**Table 1:** The prevalence of multimorbidity in community-dwelling older adults (*n* = 1,173).

Number of chronic conditions	Frequencies (*n*)	Percentages (%)
0	253	21.6
1	403	34.3
2	294	25.1
3	133	11.3
4	57	4.9
≥5	33	2.8

### 3.2 Demographic characteristics of community-dwelling older adults with multimorbidity

A total of 517 senior citizens with multimorbidity participated in the study, of whom 237were men (45.8%) and 280 women (50.4%). The average age was 73.99 ± 6.67 years, and the majority of the population was aged between 60 and 69 years (31.3%). The mean BMI was 23.57 ± 7.79 kg/m^2^ and the majority of the population had a BMI between 18.5 and 23.9 (52.2%). Most (43.1%) had primary education or less, 71.8% were married, and 42.0% had three or more children. And 47.4% of seniors worked manual jobs before retiring, and most had no religious beliefs (93.0%) or smoking habits (81.0%). Monthly personal income of 1,000–3,000 yuan ($143.9–431.7) was common (42.9%). In terms of physical exercise, more than a third (34.8%) exercised daily and almost half (48.8%) exercised within 30 min. The demographic characteristics of community-dwelling older adults with multimorbidity were demonstrated in Table 2.

**Table 2:** Demographic characteristics of community-dwelling older adults with multimorbidity (*n* = 517).

Characteristics	*n (%)*	Number of chronic conditions				*χ* ^2^	*p*
		2	3	4	≥5		
Gender						5.571	0.234
Male	237 (45.8)	144 (60.8)	53 (22.4)	22 (9.3)	18 (7.5)		
Female	280 (54.2)	150 (53.6)	80 (28.6)	35 (12.5)	15 (5.3)		
Age (years)	73.99 ± 6.67					32.644	0.001
65 ~ 69	162 (31.3)	104 (64.2)	40 (24.7)	13 (8.0)	5 (3.1)		
70 ~ 74	149 (28.8)	90 (60.4)	37 (24.8)	17 (11.4)	5 (3.4)		
75 ~ 79	99 (19.1)	56 (56.6)	21 (21.2)	15 (15.2)	7 (7.0)		
≥80	107 (20.7)	44 (41.1)	35 (32.7)	12 (11.2)	16 (15.0)		
BMI (kg/m^2^)	23.57 ± 7.79					16.652	0.163
≤18.4	33 (6.4)	18 (54.5)	8 (24.2)	3 (9.1)	4 (12.2)		
18.5 ~ 23.9	270 (52.2)	165 (61.1)	64 (23.7)	31 (11.5)	10 (3.7)		
24.0 ~ 27.9	177 (34.2)	93 (52.5)	47 (26.6)	20 (11.3)	17 (9.6)		
≥28.0	37 (7.2)	18 (48.6)	14 (37.8)	3 (8.1)	2 (5.4)		
Educational level						17.692	0.342
Elementary school and below	223 (43.1)	117 (52.5)	65 (29.1)	24 (10.8)	17 (7.6)		
Junior high school	132 (25.5)	80 (60.6)	29 (22.0)	16 (12.1)	7 (5.3)		
Technical/high school	108 (20.9)	69 (63.9)	24 (22.2)	10 (9.3)	5 (4.6)		
Diploma/bachelor degree	51 (9.9)	2 (66.7)	0 (0.0)	0 (0.0)	1 (33.3)		
Master degree and above	3 (0.6)	294 (56.9)	133 (25.7)	57 (11.1)	33 (6.3)		
Marital status						30.374	0.002
Single	4 (0.8)	0 (0.0)	2 (50.0)	1 (25.0)	1 (25.0)		
Married	371 (71.8)	225 (60.6)	88 (23.7)	35 (9.4)	33 (6.3)		
Divorced	10 (1.9)	6 (60.0)	1 (10.0)	3 (30.0)	0 (0.0)		
Widows	132 (25.5)	63 (47.7)	42 (31.8)	18 (13.6)	9 (6.9)		
Number of children						23.139	0.027
Childless	8 (1.5)	4 (50.0)	3 (37.5)	0 (0.0)	1 (12.5)		
One child	96 (18.6)	63 (65.6)	23 (24.0)	9 (9.4)	1 (1.0)		
Two children	205 (39.7)	115 (56.1)	54 (26.3)	27 (13.2)	9 (4.4)		
Three children and above	208 (40.2)	112 (53.8)	53 (25.5)	21 (10.1)	22 (10.6)		
Pre-retirement occupation						3.256	0.917
Mental labor	181 (35.0)	100 (55.2)	47 (26.0)	22 (12.2)	12 (6.6)		
Physical labor	245 (47.4)	141 (57.6)	60 (24.5)	26 (10.6)	18 (7.3)		
Unemployed	91 (17.6)	53 (58.2)	26 (28.6)	9 (9.9)	3 (3.3)		
Monthly personal income						24.918	0.071
<1,000 yuan ($143.9)	107 (20.7)	66 (61.7)	26 (24.3)	9 (8.4)	6 (5.6)		
1,000–3,000 yuan ($143.9–431.7)	222 (42.9)	132 (59.5)	53 (23.9)	24 (10.8)	13 (5.8)		
3,000–5,000 yuan ($431.7–719.5)	141 (27.3)	70 (49.6)	42 (29.8)	20 (14.2)	9 (6.4)		
5,000–10,000 yuan ($719.5–1,439)	41 (7.9)	22 (53.7)	12 (29.3)	4 (9.8)	3 (7.2)		
>10,000 yuan ($1,439)	6 (1.2)	4 (66.7)	0 (0.0)	0 (0.0)	2 (33.3)		
Religion						3.182	0.528
Yes	36 (7.0)	275 (57.2)	126 (26.2)	51 (10.6)	29 (6.0)		
No	481 (93.0)	19 (52.8)	7 (19.4)	6 (16.7)	4 (11.1)		
Smoking						6.867	0.143
Yes	98 (19.0)	237 (56.6)	116 (27.7)	42 (10.0)	24 (5.7)		
No	419 (81.0)	57 (58.2)	17 (17.3)	15 (15.3)	9 (9.2)		
Physical activity frequency						10.549	0.568
Rarely exercise	106 (20.5)	57 (53.8)	28 (26.4)	10 (9.4)	11 (10.4)		
1–2 times per week	165 (31.9)	102 (61.8)	36 (21.8)	20 (12.1)	7 (3.3)		
≥3 times per week	66 (12.8)	36 (54.5)	21 (31.8)	6 (9.1)	3 (4.6)		
Exercise every day	180 (34.8)	99 (55.0)	48 (26.7)	21 (11.7)	12 (6.6)		
Exercise duration						11.334	0.500
≤30 min	250 (48.4)	135 (54.0)	66 (26.4)	28 (11.2)	21 (8.4)		
30–60 min	227 (43.9)	133 (58.6)	54 (23.8)	28 (12.3)	12 (5.3)		
60–120 min	32 (6.2)	21 (65.6)	11 (34.4)	0 (0.0)	0 (0.0)		
≥120 min	8 (1.5)	5 (62.5)	2 (25.0)	1 (12.5)	0 (0.0)		

### 3.3 Correlation between prevalence of poor sleep quality and multimorbidity

#### 3.3.1 Comparison of the prevalence of poor sleep quality among older adults with and without multimorbidity

Overall, the prevalence of poor sleep quality with multimorbidity in older adults in the community was 57.6% (298/517). According to the Chi-square test, the difference of prevalence of poor sleep quality between senior citizens with and without multimorbidity was statistically significant (*χ*^2^ = 20.869, *p* < 0.001) (Table 3). And the prevalence of poor sleep quality in older adults with multimorbidity was 1.30 times higher than without multimorbidity (Odds Ratio = 57.6%/44.2% = 1.30).

**Table 3:** Comparison of the prevalence of poor sleep quality among older adults with different numbers of chronic conditions (*n* = 1,173).

Number of chronic conditions	With poor sleep quality	Without poor sleep quality	Total	*χ^2^*	*p*
≥2	298 (57.6)	219 (42.4)	517 (44.1)	20.869	0.000
<2	290 (44.2)	366 (55.8)	656 (55.9)		
Total	588 (50.1)	585 (49.9)	1,173		
2	158 (53.7)	136 (46.3)	30.423	0.000	
3	81 (60.9)	52 (39.1)			
4	33 (57.9)	24 (42.1)			
≥5	26 (78.8)	7 (21.2)			

#### 3.3.2 Comparison of the numbers of chronic conditions among older adults with and without poor sleep quality

Wilcoxon rank sum test results showed older adults with poor sleep quality experienced a greater number of chronic conditions than those without (*p* < 0.05) (Table 4).

**Table 4:** Comparison of the numbers of chronic conditions among older adults with and without poor sleep quality (*n* = 517).

Poor sleep quality	Number of chronic conditions (Mean ± SD)	*Z*	*p*
No (PSQI≤7)	2.56 ± 0.83	−2.301	0.021
Yes (PSQI>7)	2.78 ± 1.03		

#### 3.3.3 Prevalence of poor sleep quality by number of chronic conditions

**Figure 3:**
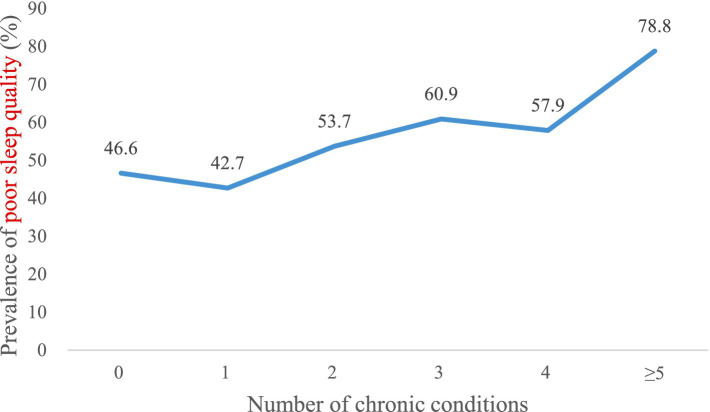
Prevalence of poor sleep quality by number of chronic conditions in the overall sample (*n* = 1,173).

**Figure 4:**
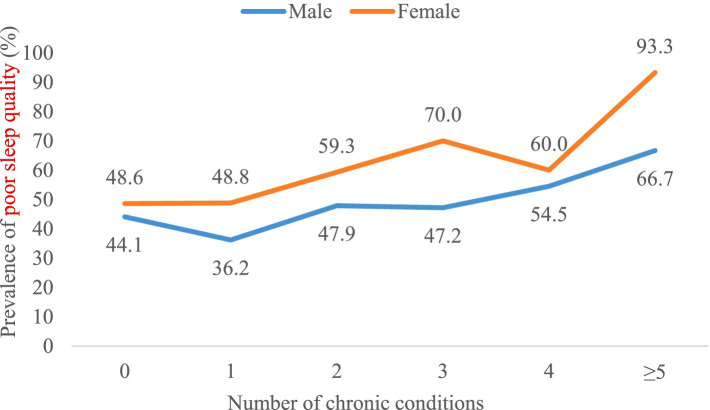
Prevalence of poor sleep quality by number of chronic conditions in sex-stratified samples (*n* = 1,173).

**Figure 5:**
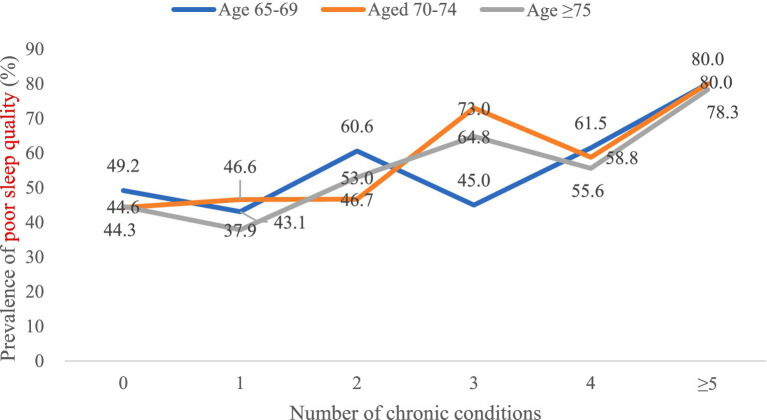
Prevalence of poor sleep quality by number of chronic conditions in age-stratified samples (*n* = 1,173).

The prevalence of poor sleep quality increased with an increasing number of chronic conditions overall (Figure 3), and women had a higher prevalence of poor sleep quality than men (Figure 4). However, there was no distinct trend in the incidence of poor sleep quality by age group (Figure 5).

### 3.4 Correlation between PSQI scores and multimorbidity

#### 3.4.1 Comparison of PSQI scores among older adults with and without multimorbidity

PSQI scores of older adults with and without multimorbidity were compared across seven PSQI components. *T*-test showed statistically significant differences in all seven PSQI components between with and without multimorbidity (*p* < 0.05) (Table 5).

**Table 5:** Comparison of the PSQI scores among older adults with and without multimorbidity (*n* = 1,173).

Multimorbidity	Subjective sleep quality	Time to fall asleep	Sleep duration	Sleep efficiency	Sleep disturbance	Hypnotic medication	Daytime function	Total scores
Yes	1.36 ± 0.77	2.70 ± 2.02	1.03 ± 1.10	1.17 ± 1.24	1.36 ± 0.61	0.26 ± 0.74	2.05 ± 1.89	7.90 ± 4.31
No	1.18 ± 0.77	2.42 ± 1.96	0.83 ± 1.06	0.99 ± 1.20	1.22 ± 0.63	0.15 ± 0.58	1.58 ± 1.74	6.73 ± 4.09
*t*	−4.050	−2.427	−3.140	−2.404	−3.621	−2.772	−4.367	−4.757
*p*	0.000	0.015	0.002	0.016	0.000	0.006	0.000	0.000

#### 3.4.2 Comparison of the PSQI scores among older adults with different numbers of chronic conditions

PSQI scores of older adults with different numbers of chronic conditions were compared across seven PSQI components. Variance analysis showed statistically significant differences in all seven components of PSQI between different numbers of chronic conditions (*p* < 0.05) (Table 6).

**Table 6:** Comparison of the PSQI scores among older adults with different numbers of chronic conditions (*n* = 517).

Number of chronic conditions	Subjective sleep quality	Time to fall asleep	Sleep duration	Sleep efficiency	Sleep disturbance	Hypnotic medication	Daytime function	Total scores
2	1.27 ± 0.75	2.51 ± 2.04	0.99 ± 1.09	1.14 ± 1.22	1.27 ± 0.61	0.20 ± 0.64	1.87 ± 1.85	7.39 ± 4.17
3	1.48 ± 0.77	2.93 ± 1.99	1.08 ± 1.09	1.10 ± 1.26	1.42 ± 0.58	0.28 ± 0.79	2.21 ± 1.90	8.33 ± 4.37
4	1.47 ± 0.78	3.15 ± 1.89	1.14 ± 1.19	1.33 ± 1.35	1.53 ± 0.66	0.33 ± 0.74	2.22 ± 1.98	8.84 ± 4.84
≥5	1.48 ± 0.77	2.76 ± 2.03	0.96 ± 1.10	1.36 ± 1.25	1.60 ± 0.58	0.40 ± 1.00	2.56 ± 1.92	9.40 ± 3.92
*F*	4.414	2.269	2.215	2.166	5.555	4.535	4.865	2.794
*p*	0.000	0.035	0.039	0.044	0.000	0.000	0.000	0.026

## 4 Discussion

### 4.1 Overall status of sleep and multimorbidity was unoptimistic

The total PSQI scores of the participants was 7.25 ± 4.23, which was much higher than the national norm in China (3.23 ± 3.12) (27). The results of this study showed that the incidence of poor sleep quality was 50.1%, which was slightly higher than a meta-analysis by Liu et al. (28) (47.2%), and significantly higher than the study by Liu et al. (29) (16.8%), and Kathleen (30) (30–40%). This may be related to the different age groups, regions and years of study, but it also reflects to some extent that the incidence of poor sleep quality among the senior citizens in China is at a high level. In this cross-sectional study, a prevalence of multimorbidity (56.2%) was observed in the study population in Hunan Province, China. This was consistent with the findings of multimorbidity in primary care in Canada (57.9%). This was found to be higher than the longitudinal study of health and retirement in China conducted by Zhang et al. (31) in 2015 (43.6%) and studies of older people in Mexico in 2018 (27.3%) (32). Similar studies from other countries reported multimorbidity of 69.4% in South Africa (33) and 62.8% in Japan (34). This variation may be explained by differences in the definition or measurement of multimorbidity, study populations, demographics, study settings, and self-reported data (35). A combination of two and three chronic conditions dominated the pattern of multimorbidity among community-dwelling older adults. With the rapid development of the society and the changes in lifestyles, chronic diseases are becoming younger and more prevalent, which not only greatly increases the difficulty of providing health care for chronic diseases, but also places a heavy economic burden on families, society and the country. Kingston et al. (36) predicted that by 2035, the number of patients with ≥ 4 chronic diseases in the UK will double from 2015, and society and families will soon face a heavy economic burden.

### 4.2 Participants with multimorbidity had a higher incidence of poor sleep quality

This study found that older adults without multimorbidity had better overall sleep quality and a lower incidence of poor sleep quality than those with multimorbidity. And the incidence of poor sleep quality was 1.30 times higher in older adults with multimorbidity than in those without. This suggests that older adults with multimorbidity in the community may be more prone to poor sleep quality. Fu et al. (37) found that chronic diseases such as hypertension and cervical spondylosis affect sleep quality in older adults. One reason for poor sleep quality in older adults with hypertension is that they are overly concerned about the cost of medical care and the effectiveness of treatment (38). Patients with heart disease have a weakened respiratory regulatory center and often experience breathing difficulties, resulting in sleep problems such as waking up easily at night and excessive daytime sleep (39). Sleep disturbance in seniors with cervical spondylosis, which causes physical pain and restricted movement, resulting in discomfort that affects sleep quality (40). Priority should therefore be given to the physical well-being of older adults in the community and to active management of chronic conditions to improve their sleep.

### 4.3 The number of chronic conditions had additive effects on poor sleep quality

In general, there has been an increasing trend in poor sleep quality as the number of chronic conditions has increased. We observed differences in the incidence of poor sleep quality between men and women. Specifically, women reported poorer sleep quality than men, which is consistent with other epidemiological studies (22). In addition, the prevalence of poor sleep quality increased with an increasing number of chronic conditions overall. That is, the greater the number of chronic conditions, the worse the sleep quality. The association with sleep problems is likely to be explained by psychological factors (e.g., higher levels of anxiety and depression), lifestyle factors (e.g., obesity, physical inactivity and high levels of sedentary time), and subclinical inflammation (21). Therapy outcomes may be worse in people with multiple chronic diseases, which may be an important factor in fatal outcomes (41). To some extent, multimorbidity may lead to sleep problems through an accumulation of different pathways (20), while sleep problems can also likely result in multimorbidity via circadian rhythm dysfunction and generalized inflammation (42).

### 4.4 Women are more likely to have poor sleep quality than men

We observed differences in the incidence of poor sleep quality between men and women. Specifically, women reported poorer sleep quality than men, which is consistent with other epidemiological studies (22). Binary logistic regression analysis showed that gender was the risk factor for poor sleep quality in older adults with multimorbidity, with women having 1.632 times the risk of men. According to the standardized regression coefficient, gender has the greatest influence on PSQI scores. Some researchers believe that women care more than men about family relationships, the future of their children, and even the details of the third generation (43). These can lead to a higher incidence of poor sleep quality in women than in men.

### 4.5 Participants with multimorbidity had the most severe difficulty falling asleep

The results of this study showed that seniors with multimorbidity had the highest sleep quality score in terms of time to fall asleep, indicating that older adults in the community had the greatest difficulty falling asleep. On the one hand, this may be related to the aging of the body, which leads to changes in the sleep rhythm. On the other hand, long periods spent in bed, reduced daytime activity and other general factors make it harder for seniors to fall asleep. We also found that hypnotic medication scored the lowest of the seven components, suggesting that the use of hypnotic medication is not widespread among older adults. This suggests that older adults should not only pay attention not only to the quality of their sleep, but also pay attention to other dimensions, such as the time it takes to fall asleep. There is evidence that seniors often go to bed (too) early and before the biological triggers that promote sleep (42).

### 4.6 Sleep and multimorbidity may interact with each other

Previous studies have shown that short or long sleep duration and poor sleep satisfaction are associated with the coexistence of multiple diseases (44), and the coexistence of sleep problems and multiple diseases may be mutually reinforcing (45). Ucar et al. (46) investigated the association between sleep duration and cardiovascular and metabolic comorbidities in patients with obstructive sleep apnea and confirmed that ensuring adequate sleep duration and modifying lifestyle factors and sleep hygiene could be beneficial in the prevention of cardiovascular and coronary events. Poor sleep quality can affect hormone levels in the body by stimulating phytomeres, thereby affecting the function of digestive organs and causing chronic digestive disorders (47). In addition, poor sleep quality leads to a reduction in functional and stimulatory connections in brain regions associated with executive function, thereby reducing an individual’s ability to regulate autonomy and affecting an individual’s level of mental health, and psychological factors are associated with many chronic diseases (48, 49). Previous studies (50) have reported the correlation between the combined effect of sleep duration and sleep quality and various chronic diseases, and the results showed that the combined effect of insufficient sleep duration and poor sleep quality had the strongest correlation with chronic diseases such as hypertension. Therefore, there is a need to understand the potential impact of poor sleep and multiple chronic comorbidities in order to inform clinical and public health strategies for the prevention and management of comorbidities. Efforts to better understand and manage multimorbidity should continue among researchers and policy makers.

### 4.7 Strengths and limitations

Our study has several strengths. First, to the best of our knowledge, this is the first large population-based study to provide evidence of a correlation between sleep and multimorbidity among older adults in the Chinese population. As the aging process accelerates and life expectancy increases, there is a need to study China’s older adults because of the significant differences in culture and health care system compared to other countries. In addition, the data collected were analyzed using reliable statistical methods, and the results may provide theoretical support for future interventions for poor sleep quality and chronic disease.

Some limitations need to be addressed. First, the PSQI only assesses sleep quality, which is often uncorrelated with objective measures of sleep. Although self-report is the most feasible method for population-based studies, it has the potential to underestimate prevalence and may lead to some misclassification. In addition, the measure cannot be used to make sleep diagnoses, but actual clinical sleep diagnoses could be included in future research. One of the limitations of this study is that its cross-sectional design does not allow us to establish a causal relationship between the variables studied and multimorbidity. For example, (a) people with mental illness or cognitive impairment were excluded and these individuals might be expected to have worse medical outcomes; (b) socio-demographics, BMI and sleep disrupting behaviors could have been used as independent correlates of sleep quality; (c) the number of related chronic diseases/conditions could have been expanded to include obesity and mental health and eating disorder diagnoses. Further studies on the pathophysiological processes underlying multimorbidity are needed to improve understanding of the interactions and synergies between different diseases.

## 5 Conclusion

Community-dwelling older adults with multimorbidity were more likely to have sleep problems. The number of chronic conditions also had an additive effect on sleep problems, and women reported poorer sleep quality than men. One of the most challenging aspects of falling asleep was for participants with multimorbidity.

## Data Availability

The original contributions presented in the study are included in the article/supplementary material, further inquiries can be directed to the corresponding author.

## References

[1] World Health Organization. (2022). Ageing and health. Available online at: . (https://www.who.int/news-room/fact-sheets/detail/ageing-and-health).

[2] National Bureau of Statistics. (2020). Statistical bulletin of National Economic and social development in 2019. Available online at: . (http://www.stats.gov.cn/xxgk/sjfb/tjgb2020/202006/t20200617_1768655.html).

[3] National Bureau of Statistics. (No. 5) (2021). Bulletin of the seventh National Population Census. Available online at: . (http://www.stats.gov.cn/tjsj/tjgb/rkpcgb/qgrkpcgb/202106/t20210628_1818824.html).

[4] Department of aging health. (2020). Interpretation of the guidance opinions on promoting the development of the elderly supplies industry. Available online at: . (http://www.nhc.gov.cn/lljks/s7786/202001/ef8f574d712946d7ae49c1e6b3407e8f.shtml).

[5] Dereje D Mossie A Abebe S. Sleep quality and its associated factors among nurses in Jimma zone public hospitals, Southwest Ethiopia, 2018. Sleep Hypn. (2020) 21:292–301. doi: 10.37133/Sleep.Hypn.2019.21.0197

[6] Tang J Liao Y Kelly BC Xie L Xiang YT Qi C. Gender and regional differences in sleep quality and insomnia: a general population-based study in Hunan Province of China. Sci Rep. (2017) 7:43690. doi: 10.1038/srep43690 28262807 PMC5337959

[7] Gothe NP Ehlers DK Salerno EA Fanning J Kramer AF Mcauley E. Physical activity, sleep and quality of life in older adults: influence of physical, mental and social well-being. Behav Sleep Med. (2020) 18:797–808. doi: 10.1080/15402002.2019.1690493 31713442 PMC7324024

[8] Koh SH Chang KJ Hong CH. Relationship between perceived low sleep quality and depression in a Korean elderly population. Eur Neuropsychopharmacol. (2019) 29:S379–80. doi: 10.1016/j.euroneuro.2018.11.580

[9] BomboisSDeramburePPasquierFMonacaC. Sleep disorders in aging and dementia. J Nutr Health Aging. (2010) 14:212–7. doi: 10.1007/s12603-010-0052-720191256

[10] Stranges S Tigbe W Gomez-Olive F Thorogood M Kandala NB. PS09 sleep problems: an emerging global epidemic? Findings from the Indepth who-sage study among over 40,000 older adults from eight countries across Africa and Asia. J Epidemiol Community Health. (2012) 66:A41–2. doi: 10.1136/jech-2012-201753.108 PMC3397790 22851813

[11] Kosari S Koerner J Naunton M. Integrating pharmacists into aged care facilities to improve the quality use of medicine (PiRACF study): protocol for a cluster randomised controlled trial. Trials. (2021) 22:1–12. doi: 10.1186/s13063-021-05335-0 34116708 PMC8193166

[12] Sim SZ Koh HL Lee SPS Young DYL Lee ES. How does multimorbidity affect middle-aged adults? A cross-sectional survey in the Singapore primary healthcare setting. BMC Fam Pract. (2020) 1:1–19. doi: 10.21203/rs.2.24538/v2 PMC7490863 32928131

[13] Wang H Zhang L Fang XY. Prevalence and spatial analysis of chronic comorbidity among Chinese middle-aged and elderly people. Chinese. Gen Pract. (2022) 25:1186–90. doi: 10.12114/j.issn.1007-9572.2022.0127

[14] Marengoni A Angleman S Melis R Mangialasche F Karp A Garmen A. Aging with multimorbidity: a systematic review of the literature. Ageing Res Rev. (2011) 10:430–9. doi: 10.1016/j.arr.2011.03.003 21402176

[15] David O Frank C Evelyn C Winston C Sandaly P Fabio P. Risk factors for chronic diseases and multimorbidity in a primary care context of Central Argentina: a web-based interactive and cross-sectional study. Int J Environ Res Public Health. (2017) 14:251. doi: 10.3390/ijerph14030251 28257087 PMC5369087

[16] Najafian J Nouri F Mohammadifard N. Association between sleep duration and hypertension: Isfahan healthy heart program, Iran. ARYA. Atherosclerosis. (2019) 15:1657. doi: 10.22122/arya.v15i1.1657 PMC6597801 31440281

[17] Chew M. NYQ. The associations of objectively measured sleep duration and sleep disturbances with diabetic retinopathy. Diabetes Res Clin Pract. (2020) 159:107967. doi: 10.1016/j.diabres.2019.107967, PMID: 31805348

[18] Zhang J Zhang J Wu H Wang R. Sleep duration and risk of hyperlipidemia: a systematic review and meta-analysis of prospective studies. Sleep Breath. (2022) 26:997–1010. doi: 10.1007/s11325-021-02504-y 34618292

[19] Ran L Chen Q Zhang J Tu X Tan X Zhang Y. The multimorbidity of hypertension and osteoarthritis and relation with sleep quality and hyperlipemia/hyperglycemia in China's rural population. Sci Rep. (2021) 11:17046. doi: 10.1038/s41598-021-96523-0 34426632 PMC8382830

[20] Smith L Shin JI Jacob L Schuch F Oh H Tully MA. Association between physical multimorbidity and sleep problems in 46 low-and middle-income countries. Maturitas. (2022) 160:23–31. doi: 10.1016/j.maturitas.2022.01.007 35550705

[21] Sabia S Dugravot A Léger D Ben HC Kivimaki M Singh-Manoux A. Association of sleep duration at age 50, 60, and 70 years with risk of multimorbidity in the UK: 25-year follow-up of the Whitehall II cohort study. PLoS Med. (2022) 19:e1004109. doi: 10.1371/journal.pmed.1004109 36256607 PMC9578599

[22] Lima MG Barros M Malta DC Medina L Szwarcwald CL. Association of self-reported sleep problems with morbidities and multimorbidities according to sex: National Health Survey 2019. Epidemiol Serv Saude. (2022) 31:e2021386. doi: 10.1590/ss2237-9622202200007.especial 35730889 PMC9897816

[23] Lin Y Hu Y Guo J Chen M Xu X Wen Y. Association between sleep and multimorbidity in Chinese elderly: results from the Chinese longitudinal healthy longevity survey (CLHLS). Sleep Med. (2022) 98:1–8. doi: 10.1016/j.sleep.2022.06.007 35753186

[24] Xue B Xue Y Dong F Zheng X Shi L Xiao S. The impact of socioeconomic status and sleep quality on the prevalence of multimorbidity in older adults. Front Public Health. (2022) 10:959700. doi: 10.3389/fpubh.2022.959700 36225792 PMC9548700

[25] Zhenqiu S Yongyong X. Medical statistics. Beijing: People 's Medical Publishing House (2014).

[26] Buysse DJ Rd C Monk TH Berman SR Kupfer DJ. The Pittsburgh sleep quality index: a new instrument for psychiatric practice and research. Psychiatry Res. (1989) 28:193–213. doi: 10.1016/0165-1781(89)90047-4 2748771

[27] Liu X Tang M. Reliability and validity of the Pittsburgh sleep quality index. Chin J Psychiatry. (1996) 29:5. doi: 10.1007/BF02951625

[28] Liu Y Dong Y Li X Mao XI Peng G Liu L. Meta-analysis of the prevalence of sleep disorder among Chinese elderly aged 60 years and over. Modern. Prev Med. (2014) 41:5.

[29] Xiujun L Yuanyuan L Benna Z Yanping R. Sleep statusand related factors of residents over 16 years old inTongzhou district of Beijing. Med J Chin People Health. (2020) 32:41. doi: 10.3969/j.issn.1672-0369.2020.10.041

[30] Kathleen Y. Sleep disorders in the elderly. Clin Geriatr Med. (2018). doi: 10.1016/j.cger.2018.01.008 29661333

[31] Ran Z Yun L Shan-shan Z. Prevalence pattern and component correlation of chronic disease comorbidity among the elderly in China. Chin J Public Health. (2019) 35:3. doi: 10.11847/zgggws1120351

[32] Horacio IG Eduardo MC de Lourdes MM de la Rosa-Santillana R Ángel FM José VJ. Prevalence of multimorbidity in subjects aged ≥60 years in a developing country. Clin Interv Aging. (2018) 13:1129–33. doi: 10.2147/CIA.S154418 29942121 PMC6005321

[33] Chang AY Gómez-Olivé FX Payne C Rohr JK Manne-Goehler J Wade AN. Chronic multimorbidity among older adults in rural South Africa. BMJ Glob Health. (2019) 4:e001386. doi: 10.1136/bmjgh-2018-001386 PMC6688670 31423345

[34] Aoki T Yamamoto Y Ikenoue T Onishi Y Fukuhara S. Multimorbidity patterns in relation to polypharmacy and dosage frequency: a nationwide, cross-sectional study in a Japanese population. Sci Rep. (2018) 8:3806. doi: 10.1038/s41598-018-21917-6 29491441 PMC5830504

[35] Asogwa OA Boateng D Marzà-Florensa A Peters S Levitt N van Olmen J. Multimorbidity of non-communicable diseases in low-income and middle-income countries: a systematic review and meta-analysis. BMJ Open. (2022) 12:e49133. doi: 10.1136/bmjopen-2021-049133 PMC8785179 35063955

[36] Kingston A Robinson L Booth H. Projections of multi-morbidity in the older population in England to 2035: estimates from the population ageing and care simulation (PACSim) model. Age Ageing. (2018) 47:374–80. doi: 10.1093/ageing/afx201, PMID: 29370339 PMC5920286

[37] Liting F Ruixue M Shanshan Z Qianqian A. Multiple logistic regression analysis of influencing factors of sleep quality in the elderly. Chin J Gerontol. (2022) 42:462–5. doi: 10.3969/j.issn.1005-9202.2022.02.052

[38] Yujuan W Lianzhao Y Ling C. Research on the status quo of sleep quality and anxiety in elderly patients with hypertension in the community. Chin Nurs Res. (2018) 32:4. doi: 10.12102/j.issn.1009-6493.2018.09.033

[39] Kazuomi K Pickering TG Yuji U Satoshi H Yoko H Masato M. Morning surge in blood pressure as a predictor of silent and clinical cerebrovascular disease in elderly hypertensives: a prospective study. Circulation. (2019) 107:1401–6. doi: 10.1161/01.cir.0000056521.67546.aa 12642361

[40] Xxiuqing L. Analysis of sleep disorders in patients with cervical spine syndrome and Mongolian medicine care. Nei Mongol J Trad Chin Med. (2014) 33:2. doi: 10.3969/j.issn.1006-0979.2014.23.146

[41] ZhangLSunFLiYTangZMaL. Multimorbidity in community-dwelling older adults in Beijing: prevalence and trends, 2004–2017. J Nutr Health Aging. (2020) 25:1–4. doi: 10.1007/s12603-020-1467-433367471

[42] Brown RF Thorsteinsson EB. Comorbidity: Symptoms, conditions, behavior and treatments. New York, NY: Springer (2020).

[43] Sharma P Maurya P. Gender differences in the prevalence and pattern of disease combination of chronic multimorbidity among Indian elderly. Ageing Int. (2022) 47:265–83. doi: 10.1007/s12126-021-09419-9

[44] Nicholson K Rodrigues R Anderson KK Wilk P Guaiana G Stranges S. Sleep behaviours and multimorbidity occurrence in middle-aged and older adults: findings from the Canadian longitudinal study on aging (CLSA). Sleep Med. (2020) 75:156–62. doi: 10.1016/j.sleep.2020.07.002 32858355

[45] Sakib MN Shooshtari S St John P Menec V. The prevalence of multimorbidity and associations with lifestyle factors among middle-aged Canadians: an analysis of Canadian longitudinal study on aging data. BMC Public Health. (2019) 19:243. doi: 10.1186/s12889-019-6567-x 30819126 PMC6394050

[46] Ucar ZZ Cirak AK Olcay S Uysal H Demir AU Zacar RF. Association of Duration of sleep and cardiovascular and metabolic comorbidities in sleep apnea syndrome. Sleep Disord. (2012) 2012:316232. doi: 10.1155/2012/316232 23471129 PMC3581129

[47] Verweij IM Romeijn N Smit DJ Piantoni G. Sleep deprivation leads to a loss of functional connectivity in frontal brain regions. BMC Neurosci. (2014) 15:1–10. doi: 10.1186/1471-2202-15-88 25038817 PMC4108786

[48] Orr WC Fass R Sundaram SS Scheimann AO. The effect of sleep on gastrointestinal functioning in common digestive diseases. Lancet Gastroenterol Hepatol. (2020) 5:616–24. doi: 10.1016/S2468-1253(19)30412-1 32416862

[49] Ciro C. Common psychological factors in chronic diseases. Front Psychol. (2019) 10:2727. doi: 10.3389/fpsyg.2019.02727 31866912 PMC6909152

[50] Yan W Hao M Yan-Rui J. Relationship between duration of sleep and hypertension in adults: a Meta-analysis. Journal of clinical sleep medicine: JCSM: official publication of the American Academy of. Sleep Med. (2015) 11:1047–56. doi: 10.5664/jcsm.5024 PMC4543249 25902823

